# Supplementary material for: Heart Disease and Arboviruses: A Systematic Review and Meta-Analysis
Source: Viruses. 2022 Sep 8;14(9):1988. doi: 10.3390/v14091988 (PMC9502577; doi:10.3390/v14091988)
Supplement: Supplementary file 1 [file viruses-14-01988-s001.zip › viruses-1853148-supplementary.pdf]

Figure S1. Funnel plot for evaluation of publication bias with pseudo 95% confidence limits (Begg's test)

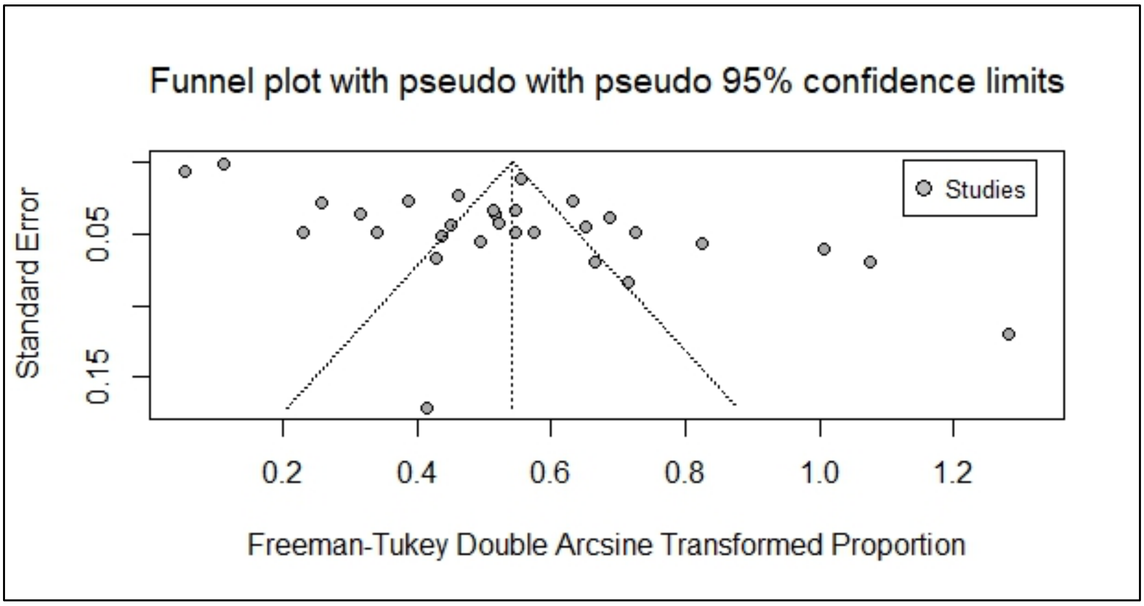

Figure S2. Funnel plt with psudo 95% confidence limits after trim fill adjustment (Egger's test)

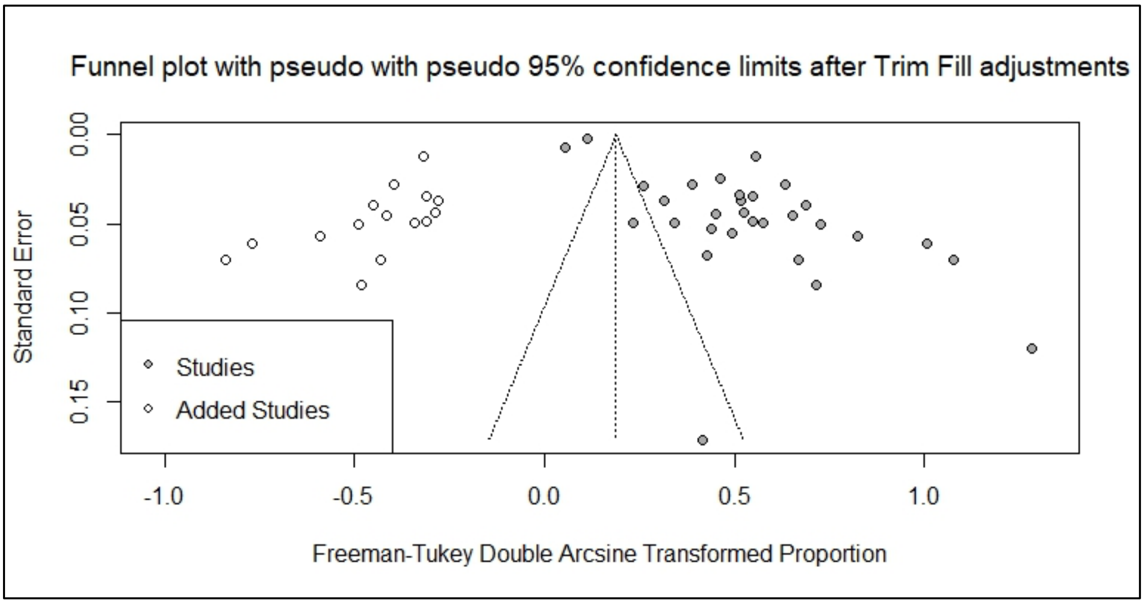

Figure S3. Forest plot comparison of frequency of cardiac events in patients with Dengue, grouped by country group.

Studies	Events	Total	(%)	CI 95%	Weight
**India**					
Arora, 2015	44	120	36.67	[28.06; 45.95]	9.7%
Girdhar, 2017	57	211	27.01	[21.15; 33.54]	11.0%
Yadav, 2017	28	104	26.92	[18.69; 36.51]	9.4%
Kalakutakar, 2019	44	100	44.00	[34.08; 54.28]	9.2%
Bhatt, 2020	44	182	24.18	[18.15; 31.06]	10.7%
Sha, 2020	112	320	35.00	[29.78; 40.50]	11.7%
Gupta, 2022	41	150	27.33	[20.38; 35.20]	10.3%
**Subtotal**	**370**	**1187**	**31.17**	**[26.34; 36.22]**	**71.9%**
Heterogeneity: $Tau^2 = 0.0035$; $Chi^2 = 18.81$, df = 6 ($P < 0.01$); $I^2 = 68\%$					
**Thailand**					
Thanachartwet, 2016	65	162	40.12	[32.51; 48.10]	10.4%
Mansanguan, 2021	18	81	22.22	[13.73; 32.83]	8.6%
Khongphatthanayothin, 2007	16	91	17.58	[10.40; 26.98]	9.0%
**Subtotal**	**99**	**334**	**26.49**	**[14.24; 40.88]**	**28.1%**
Heterogeneity: $Tau^2 = 0.0155$; $Chi^2 = 17.12$, df = 2 ($P < 0.01$); $I^2 = 88\%$					
**Total**	**469**	**1521**	**30.00**	**[25.12; 35.13]**	**100.0%**
Heterogeneity: $Tau^2 = 0.0058$; $Chi^2 = 36.35$, df = 9 ($P < 0.01$); $I^2 = 75\%$					
Test for subgroup differences: $Chi^2 = 0.38$, df = 1 ($P = 0.54$)					

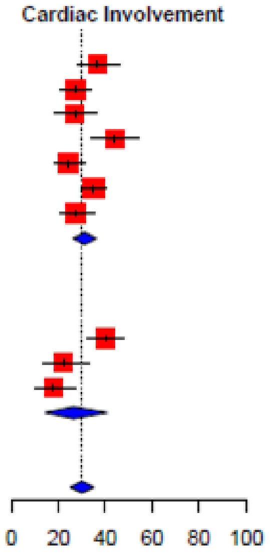

Table S1. Methodological quality assessment by the NOS Score

**Author Year Journal**	**Arbovirus**	**Selection**				**Comparability**	**Outcome/**			
		Representativeness of the exposed cohort	Selection of the non exposed cohort	Ascertainment of exposure	Demonstration that outcome of interest was not present at start of study	Comparability of cohorts on the basis of the design or analysis	Assessment of outcome	Was follow-up long enough for outcomes to occur	Adequacy of follow up of cohorts	**Total Score/Quality assessment**
Lakshman et al. 2018 Tropical Doctor	DENV[#]	--	--	*	--	--	*	*	*	4/ Poor
Arora et al. 2015 JAPI	DENV	--	--	*	*	**	*	--	*	6/ Fair
Bhatt et al. 2020 Infection	DENV	--	--	*	*	**	*	--	*	6/ Fair
Bonifay et al. 2018 PLoS ONE	CHIKV[##]	--	--	*	*	--	*	--	*	4/ Poor
Butunbatu et al. 2019 Journal of Tropical Pediatrics	DENV	---	---	*	*	**	*	--	*	6/ Fair
Cabrera-Rego et al. 2021 Enferm Infec. Microb Clin	DENV	--	--	*	*	**	*	*	*	7/ Good
Sha et al. 2020 International Journal of Cardiology	DENV	--	--	*	*	**	*	*	*	7/ Good
Li et al. 2016 Medicine (Baltimore)	DENV	--	--	--	*	**	*	*	*	6/ Fair
La-Orkhun et al. 2011 Annals of Tropical Paediatrics	DENV	--	--	*	--	--	*	*	*	4/ Poor
Kirawittaya et al. 2015 PLoS NT	DENV	--	*	*	*	*	*	*		6/ Fair
Salgado et al. 2010 *Pediatr Infect Dis*	DENV	--	--	*	--	*	*	*	*	5/ Poor
Yacoub et al. 2017 PLoS NTD	DENV	--	--	*	--	*	*	*	*	5/ Fair
Thanachartwet et al. 2016	DENV	*	--	*	--	**	*	*	*	7/ Good

PLoS One										
Khongphatthanayothin et al. 2007 Pediatr Crit Care Med	DENV	--	--	*	*	**	*	*	*	7/ Good
Cavalcanti et al. 2017 PLoS One	ZKV###	--	--	*	*	*	*	*	*	6/ Fair
Pothapregada et al. 2026 Indian J Pediatr	DENV	--	--	*	--		*	*	*	4/ Poor
Saldarriaga et al. 2013 Revista Colombiana de Cardiología	DENV	--	--	*	--	--	*	--	*	3/ Poor
Hussain et al. 2016 P J M H S	DENV	--	--	*	*	*	*	*	*	6/ Fair
Kumar et al. 2013 Pediatr Cardiol	DENV			*	*	**	*	--	*	6/ Fair
Khositseth et al. 2012 Journal of Pediatric Intensive Care	DENV	--	--	*	--	--	*	*	*	4/ Poor
Khongphatthanayothin et al. 2003 Intensive Care Med	DENV	--	--	*	*	*	*	*	*	6/ Fair
Godoy et al. 2018 Pediatr. (Asunción)	DENV	--	--	*	*	*	*	*	*	6/ Fair
Kabra et al. 1998 The national medical journal of India	DENV	--	--	--	*	--	*	*	*	4/ Poor
Satarasinghe et al. 2007 British Journal of Cardiology	DENV	--	--	*	*	*	*	*	*	6/Fair
Obeyesekere et al. 1973 American Heart Journal	CHIKV		*	*	?	?	*	*	*	5/ Poor
Wali et al. 1998 International Journal of Cardiology	DENV	--	--	*	--	--	*	*	*	4/ Poor
Weerakoon et al. 2011 BMC Research	DENV	--	--	*	--	--	*	--	*	3/ Poor
Lee et al. 2021 Journal of Microbiology, Immunology and Infection	DENV	--	--	*	--	--	*	*	*	4/ Poor
Yadav et al. 2017 J. Evolution Med. Dent. Sci	DENV	--	--	*	*	**	*	?	*	6/ Fair

Lee et al. 2022 Travel Medicine and Infectious Disease	DENV	--	--	*	*	*	*	*	*	6/Fair
Wei et al. 2022 PLos NTD	DENV	--	*	*	--	--	*	*	*	5/Fair
Mansanguan et al. 2021 *BMC Infect Dis*	DENV	*	--	*	*	**	*	*	*	8/ Good
Kalkutakar et al. 2019 International Journal of Current Microbiology and Applied Sciences	DENV	--	--	*	*	**	*	--	*	6/ Fair
Miranda et al. 2013 Clinical Infectious Diseases English	DENV	--	--	*	--	--	*	*	*	4/ Poor
Girdhar et al. 2017 Journal of Medibal Science and Clinical Searchch	DENV	--	--	*	*	**	*	--	*	6/ Fair
Wichmann et al. 2009 Southeast Asian J Trop Med Public Health	DENV	--	--	*	--	--	*	--	*	3/ Poor
Gupta et al. 2022 Tropical Doctor English	DENV	--	--	*	*	*	*	*	*	6/Fair

Legend: # Dengue Virus; ## Chikungunya Virus; ### Zika Virus

Table S2. Meta-regression of the variables gender and clinical severity of Dengue in cardiac outcome

Variables studied	N° of Studies	N° of Patients	CI	p-value
**Sex**				
Female	17	1,145	(-0.0037 - 0.0029)	0.802
Male	17	1,493	(-0.0043 - 0.0035)	0.833
**Clinical Classification of Dengue**				
Dengue Fever	10	2,284	(-0.0001 - 0.0005)	0.300
Dengue Fever Severe	14	1,097	(-0.0035 - 0.0007)	0.194
**Death associated with cardiac events**	19	70,485	(-0.0062 - 0.0491)	0.128
**Publication Year**	19	70,485	(-0.0375 - 0.0070)	***0.004***
